# Multi-Omics Integration Uncovers That Tenofovir Disoproxil Fumarate Is Linked to Hepatic Metabolic Reprogramming Independent of Viral Infection

**DOI:** 10.3390/life16061017

**Published:** 2026-06-17

**Authors:** Yuanqin Duan, Yunling Xue, Jing Tang, Teng Long, Zhiwei Chen, Mingli Peng, Peng Hu

**Affiliations:** The Key Laboratory of Molecular Biology for Infectious Diseases, Department of Infectious Diseases, Institute for Viral Hepatitis, Chinese Ministry of Education, The Second Affiliated Hospital of Chongqing Medical University, 76# Linjiang Road, Yuzhong District, Chongqing 400010, China

**Keywords:** tenofovir disoproxil fumarate, metabolism, CHB

## Abstract

Background and Aims: TDF is a first-line antiviral for CHB with pleiotropic effects including immunomodulation and fibrosis regression, but its virus-independent mechanisms are unclear. This study delineates TDF’s direct molecular and metabolic landscape in vivo using multi-omics. Methods: Wild-type mice received TDF or vehicle for 4 months. Liver tissues underwent RNA-seq and targeted metabolomics, followed by integrative systems biology. Results: TDF caused no hepatotoxicity but induced transcriptomic reprogramming: broad upregulation of immune/inflammatory pathways and suppression of metabolic pathways. Metabolomics confirmed perturbations in amino acid and fatty acid homeostasis. Multi-omics revealed coordinated downregulation of arginine/proline, alanine/aspartate/glutamate, and phenylalanine metabolism, restricting fibrogenic amino acids. TDF also suppressed the TCA cycle (downregulating Idh, Sdh, and Mdh), suggesting a metabolic bottleneck that was associated with paradoxically accumulated succinate and oxoglutarate—immunomodulatory danger signals. Conclusions: This first integrated atlas shows TDF actively remodels the hepatic microenvironment independent of viral infection, bridging metabolic suppression with immune activation. These findings provide an immunometabolic framework that offers new perspectives for understanding the clinical application of TDF and identifies potential biomarkers for CHB therapy. explaining TDF’s clinical superiority and identifying potential biomarkers for CHB therapy.

## 1. Introduction

Tenofovir disoproxil fumarate (TDF), an esterified prodrug of the nucleotide analog tenofovir, is currently a cornerstone of antiviral therapy for chronic hepatitis B (CHB). Recommended as a first-line agent by major international guidelines, TDF potently inhibits hepatitis B virus (HBV) DNA replication while maintaining a high barrier to viral resistance [1,2,3,4]. Extensive clinical evidence has demonstrated that sustained viral suppression achieved by TDF is strongly associated with the regression of hepatic fibrosis and a significantly reduced risk of hepatocellular carcinoma (HCC) [5,6]. However, since current analog therapies rarely achieve a complete functional cure of HBV, lifelong administration is frequently required. This necessitates a comprehensive understanding of the long-term, systemic impacts of these agents beyond their primary antiviral efficacy.

While TDF and entecavir (ETV), a potent nucleoside analog, share comparable efficacy in suppressing HBV reverse transcriptase, emerging evidence suggests that TDF exerts distinct, virus-independent pharmacological effects. Recent studies indicate that TDF actively modulates the host immune response and hepatic microenvironment [7]. For instance, Murata et al. demonstrated that TDF directly induces the production of interferon (IFN)-λ3, a cytokine with profound immunomodulatory properties [8]. Furthermore, TDF has been shown to ameliorate liver fibrosis by regulating the activation and apoptosis of hepatic stellate cells (HSCs) [9,10]. Beyond immunology and fibrosis, TDF may have a considerable impact on profoundly influences host metabolism; it modulates lipid homeostasis via the activation of the hepatic CD36/PPARα pathway [11], and exhibits a metabolic signature distinctly different from that of tenofovir alafenamide (TAF), another tenofovir prodrug [12,13,14]. These pleiotropic properties offer intriguing new perspectives on the secondary therapeutic benefits and potential metabolic side effects of TDF. Nevertheless, the global molecular mechanisms driving these extra-antiviral effects remain poorly defined, underscoring the need for further systematic exploration.

To decipher the complex molecular networks underlying these pleiotropic effects, unbiased high-throughput multi-omics approaches are indispensable. The integration of transcriptomics and metabolomics provides a robust framework to bridge the gap between gene expression profiles and ultimate phenotypic metabolic states, thereby facilitating the identification of key functional genes and metabolic pathways across various physiological conditions [14,15,16]. Applying these integrated technologies in a virus-free model allows for the precise isolation of a drug’s direct pharmacological properties from the confounding variables of viral infection and subsequent immune clearance.

To our knowledge, this is the first study to employ an integrated transcriptomic and metabolomic approach to delineate the global molecular and metabolic landscape induced by TDF administration in vivo. By utilizing wild-type mice, this study aims to uncover the direct, virus-independent pharmacological mechanisms of TDF. Our findings are expected to elucidate the molecular basis of TDF’s pleiotropic effects, thereby providing a critical theoretical foundation for optimizing CHB therapeutic strategies and offering novel insights into the extrahepatic and metabolic impacts of long-term nucleotide analog therapy (the research workflow is outlined in Supplementary Figure S1).

## 2. Materials and Methods

### 2.1. Chemicals and Animal Experiments

Tenofovir disoproxil fumarate (TDF, purity ≥ 99.5%) was generously provided by Guangshengtang Co., Ltd. (Fuzhou, China). All animal experimental procedures were approved by the Institutional Animal Care and Use Committee (IACUC) of Chongqing Medical University (Chongqing, China) under approval code No. 2024(293) on 26 February 2024, and were conducted strictly in accordance with institutional and national guidelines for animal welfare. The specific experimental protocols have been previously described [17]. Briefly, twenty-four 8-week-old female wild-type C57BL/6J littermates were randomly assigned to either the TDF group (n = 12) or the Control group (n = 12). Mice received TDF at a dose of 4.5 mg/kg/day or an equivalent volume of vehicle via oral gavage for 4 consecutive months. Mice received TDF at 45.5 mg/kg/day or an equivalent volume of vehicle via oral gavage for 4 consecutive months. This dose was selected based on our previous study [17]. According to the Reagan-Shaw formula for species dose conversion, this dose corresponds to a human equivalent dose of approximately 3.7 mg/kg/day (about 222 mg/day for a 60 kg adult), which is close to the standard clinical dose of 300 mg/day. At the experimental endpoint, peripheral blood and liver tissues were harvested for subsequent histological, biochemical, and multi-omics analyses.

### 2.2. Biochemical and Histological Assessments

Serum alanine aminotransferase (ALT) levels were quantified using an automated biochemical analyzer at the Clinical Laboratory of the Second Affiliated Hospital, Chongqing Medical University. For histological evaluation, liver tissues were promptly fixed in 4% paraformaldehyde, embedded in paraffin, and sectioned. The sections were stained with hematoxylin and eosin (H&E) according to standard protocols and visualized under a light microscope (Nikon, Tokyo, Japan) to assess pathological changes.

### 2.3. Transcriptomic Profiling

Total RNA was extracted from mouse liver tissues using TRIzol^®^ Reagent (Invitrogen, Carlsbad, CA, USA). RNA concentration, purity, and integrity were rigorously evaluated using a 2200 Bioanalyzer (Agilent Technologies, Santa Clara, CA, USA). High-quality RNA was subsequently sequenced on the DNBseq platform by BGI (Shenzhen, China). The raw sequencing data have been deposited in the NCBI Sequence Read Archive (SRA) database under the accession number PRJNA763152. Following the filtering of raw data to obtain clean reads as previously detailed [17], downstream analyses were performed. Differentially expressed genes (DEGs) were identified using the “DEGseq” R package, defined by an adjusted *p*-value (Q-value) < 0.05 and |log2(fold change)| ≥ 1. Functional enrichment analyses of the DEGs, including Gene Ontology (GO) and Kyoto Encyclopedia of Genes and Genomes (KEGG) pathways, were executed using the DAVID database and the Metascape platform. Gene Set Enrichment Analysis (GSEA) for the entire annotated gene set was performed using GSEA software (v4.2.3). Further comprehensive details are provided in the Supplementary Materials (Transcriptomic analysis).

### 2.4. Targeted Metabolomic Analysis

To ensure strict multi-omics concordance, aliquots of the identical liver tissues used for transcriptomic sequencing were subjected to targeted metabolomic analysis. Metabolite quantification was performed using an ultra-performance liquid chromatography coupled to tandem mass spectrometry (UPLC-MS/MS) system (ACQUITY UPLC-Xevo TQ-S, Waters Corp., Milford, MA, USA). Raw data generated by the UPLC-MS/MS were processed utilizing QuanMET software (v2.0, Metabo-Profile, Shanghai, China) for peak integration, calibration, and absolute quantification of metabolites. Differential metabolites (DMs) were identified based on a Variable Importance in Projection (VIP) score > 1 derived from multivariate statistical analyses, and a *p*-value < 0.05 from univariate statistical analyses. Metabolite set enrichment analysis (incorporating SMPDB and predicted sets) and KEGG pathway mapping of the DMs were conducted using MetaboAnalyst 5.0 (https://www.metaboanalyst.ca/) (accessed on 1 October 2024). Pathways with *p* < 0.05 and containing at least two mapped metabolites were considered statistically significant. Metabolite profiling and associated data analyses were executed in collaboration with Metabo-Profile Biotechnology Co., Ltd. (Shanghai, China). Additional methodological details are available in the Supplementary Materials (Metabolomic analysis).

### 2.5. Integrative Analysis of Transcriptomics and Metabolomics

To elucidate the systems-level impact of TDF, an integrative enrichment analysis was performed on the intersection of the transcriptomic and metabolomic datasets. Overlapping KEGG pathways significantly enriched in both omics layers were identified. Significance was defined by a normalized *p*-value < 0.05 from the metabolomic GSEA and a *p*-value < 0.05 from the transcriptomic enrichment. Pathway-level network visualization and molecular mapping were constructed utilizing the KEGG Mapper tool (https://www.kegg.jp/kegg/mapper/color.html) (accessed on 1 October 2024).

### 2.6. Statistical Analysis

Quantitative data are presented as the mean ± standard error of the mean (SEM). The assumption of data normality was assessed using the Shapiro–Wilk test prior to comparative analysis. For comparisons between two independent groups, statistical significance was determined using the unpaired Student’s *t*-test for normally distributed variables, or the Mann–Whitney U test for non-parametric data. All statistical analyses were performed using GraphPad Prism 8 software (GraphPad Software Inc., San Diego, CA, USA). A two-sided *p*-value < 0.05 was considered statistically significant.

## 3. Results

### 3.1. Long-Term TDF Administration Does Not Alter Basal Phenotypic Parameters in Wild-Type Mice

To evaluate the virus-independent effects of TDF and simulate long-term clinical exposure, wild-type C57BL/6J mice were administered TDF via oral gavage for 4 consecutive months. Following the treatment period, no overt signs of toxicity were observed. Quantitative assessments revealed no significant differences between the TDF and Control groups regarding liver weight (LW, Supplementary Figure S3A), body weight (BW, Supplementary Figure S3B), or the hepatic index (LW/BW, Supplementary Figure S3C). Furthermore, serum ALT levels—a primary indicator of hepatocellular injury—remained unchanged (Supplementary Figure S3D), and histological examination via H&E staining displayed normal hepatic architecture with no evidence of inflammation or steatosis in either cohort (Supplementary Figure S3E). Collectively, these findings establish that long-term TDF administration at the utilized dosage does not induce apparent hepatotoxicity or alter the basal phenotypic parameters in wild-type mice.

### 3.2. Hepatic Transcriptomic Profiling Reveals Extensive TDF-Induced Gene Expression Alterations

**Figure 1:**
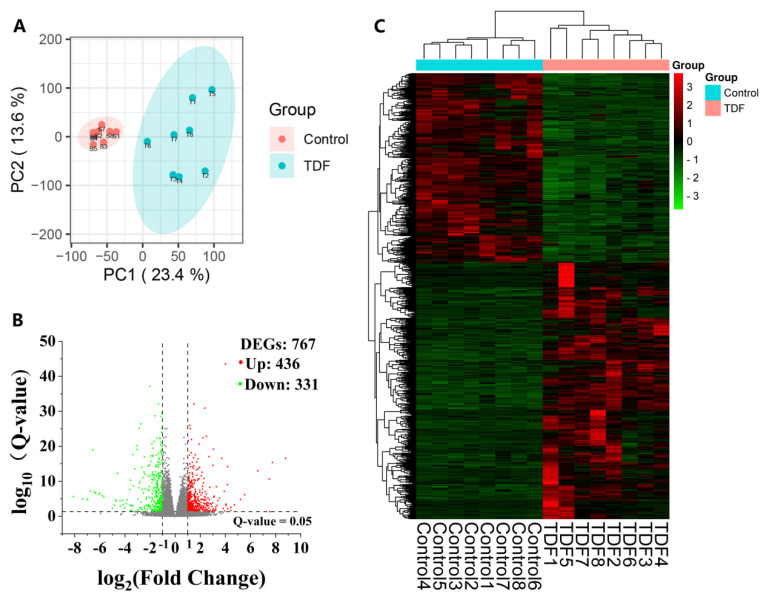
TDF-induced significant alterations of gene expression in the liver of wild-type mice. TDF induced significant alterations of gene expression in the liver of wild-type mice. (**A**) Principal component analysis (PCA) was performed with RNA-Seq data (n = 8). (**B**) The 767 significantly differentially expressed genes (DEGs) induced by TDF were visualized by the volcano plot, in which 436 genes were upregulated (red) and 331 genes were downregulated (green). (**C**) Hierarchical clustering heatmap of the 767 DEGs.

To elucidate the direct molecular impact of TDF on the liver, transcriptomic sequencing was performed. Principal component analysis (PCA) demonstrated a clear segregation between the TDF and Control groups, indicating substantial and reproducible TDF-induced transcriptional shifts within the liver (Figure 1A). Of the 17,745 annotated genes identified, 767 were recognized as differentially expressed genes (DEGs) based on the stringent criteria of an adjusted *p*-value (Q-value) < 0.05 and |log2(fold change)| ≥ 1. This comprised 436 upregulated and 331 downregulated genes in the TDF cohort (Figure 1B, Supplementary Table S1). A hierarchical clustering heatmap further visualized the distinct gene expression signatures characterizing the two groups (Figure 1C). These data robustly demonstrate that TDF actively co-occurs with and drives significant transcriptomic reprogramming in the virus-free liver.

### 3.3. TDF Transcriptionally Upregulates Immune Responses and Broadly Downregulates Metabolic Pathways

**Figure 2:**
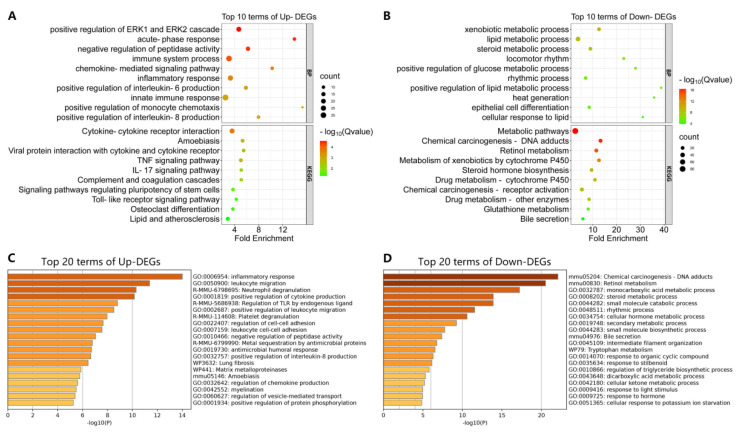
Functional enrichment analyses of the 767 differentially expressed genes (DEGs) via DAVID and Metascape. Functional enrichment analyses of the 767 differentially expressed genes (DEGs) via DAVID (**A**,**B**) and Metascape (**C**,**D**). Enrichment analysis was performed with the 436 upregulated (**A**,**C**) and 331 downregulated (**B**,**D**) DEGs. The top significantly enriched terms are shown, according to the Q-value (**A**,**B**) or *p*-value (**C**,**D**) in order. Q-value: adjusted *p*-value, i.e., false discovery rate (FDR); BP: biological process; KEGG: Kyoto Encyclopedia of Genes and Genomes.

To decode the biological significance of the 767 DEGs, functional enrichment analyses were conducted. Gene Ontology (GO) and KEGG mapping revealed a striking dichotomy: upregulated DEGs were predominantly enriched in immune and inflammatory responses—involving diverse immune cell activation and cytokine signaling (Figure 2A,C)—whereas downregulated DEGs were overwhelmingly mapped to metabolic processes, notably lipid, glucose, and retinol metabolism (Figure 2B,D; Supplementary Table S2).

**Figure 3:**
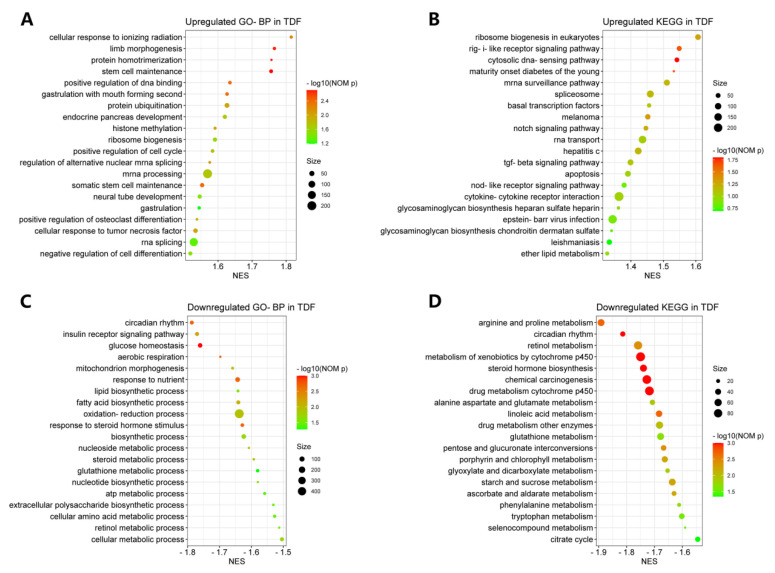
Functional enrichment analyses of all 17,745 annotated genes via Gene Set Enrichment Analysis (GSEA). Functional enrichment analyses of all 17,745 annotated genes via Gene Set Enrichment Analysis (GSEA). Upregulated biological processes (**A**) and KEGG pathways (**B**) induced by TDF. Downregulated biological processes (**C**) and KEGG pathways (**D**) induced by TDF. The top 20 significantly enriched gene sets are shown, according to the NES in order. NES: normalized enrichment score. NOM *p*: normalized *p*-value.

This transcriptomic shift was further corroborated by Gene Set Enrichment Analysis (GSEA) across the entire transcriptome. TDF administration led to the upregulation of 674 biological processes (44 significant) and 157 pathways (4 significant), which were broadly associated with host defense, viral infection responses, and nucleic acid/protein modification (Figure 3A,B). Conversely, TDF significantly downregulated 249 biological processes (23 significant) and 79 pathways (20 significant) that were highly specific to fundamental metabolic networks, including amino acid, lipid, nucleotide, and carbohydrate metabolism (Figure 3C,D; Supplementary Table S3). In summary, the transcriptomic landscape indicates that TDF skews the hepatic microenvironment toward immune activation while simultaneously suppressing core metabolic functions. In summary, the transcriptomic landscape reveals that TDF exposure is associated with an immune-activated hepatic microenvironment, together with reduced expression of core metabolic pathways.

### 3.4. Targeted Metabolomics Delineates TDF-Induced Shifts in the Hepatic Metabolome

To validate the downstream phenotypic consequences of the observed transcriptional metabolic suppression, targeted UPLC-MS/MS metabolomics was performed on the corresponding liver tissues. A total of 220 high-confidence metabolites across 16 biochemical classes were quantified (Supplementary Table S4). The baseline metabolome was dominated by fatty acids (21.82% by count; 57.28% by abundance), amino acids (20.00% by count; 17.16% by abundance), and nucleotides (20.03% by abundance) (Supplementary Figure S4A–C).

**Figure 4:**
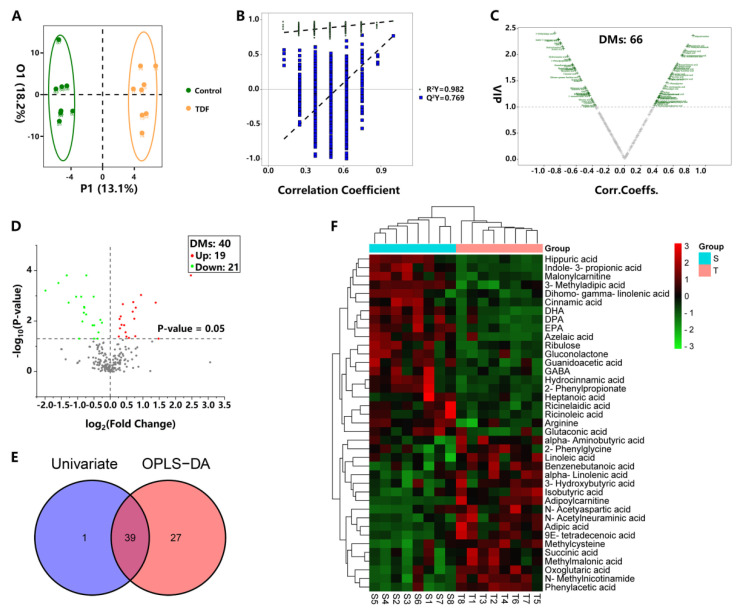
TDF-associated alterations of metabolite accumulation in the liver of wild-type mice. TDF induced significant alterations in metabolite accumulation in the liver of wild-type mice. (**A**) Orthogonal partial least squares-discriminant analysis (OPLS-DA) score plot. (**B**) OPLS-DA model validation. The permutation test with 1000 iterations was performed, and then this OPLS-DA model was valid without overfitting (R^2^X = 0.313, R^2^Y = 0.982, Q^2^Y = 0.769). (**C**) Volcano plot of OPLS-DA model, VIP > 1 was used to identify candidate differential metabolites (DMs). VIP: variable importance in projection, the strength of both variable contribution; Corr.Coeffs: correlation coefficients between every single metabolite with the first principal component. (**D**) Volcano plot of univariate statistics, threshold value for significant DM selection was *p*-value < 0.05 and |log2FoldChange| ≥ 0. (**E**) Venn diagram of the significant DMs from OPLS-DA model and univariate statistics. (**F**) Hierarchical clustering heatmap of the 39 union DMs.

Multivariate orthogonal partial least squares-discriminant analysis (OPLS-DA) yielded a distinct separation between the TDF and Control groups (Figure 4A). The model exhibited high robustness and predictive capacity (R^2^Y = 0.982, Q^2^Y = 0.769; Figure 4B). Using a Variable Importance in Projection (VIP) threshold > 1.0, 66 metabolites were flagged as critical discriminants (Figure 4C, Supplementary Table S6A). Concurrent univariate analysis (*p* < 0.05) identified 40 differential metabolites (DMs) (Figure 4D, Supplementary Table S6B). The intersection of these statistical approaches yielded 39 robust DMs (18 upregulated and 21 downregulated) that strictly characterized the TDF-treated liver (Figure 4E, Supplementary Table S7). Hierarchical clustering of these 39 DMs confirmed highly divergent metabolic profiles between the cohorts (Figure 4F).

### 3.5. Metabolomic Enrichment Highlights Profound Alterations in Amino Acid and Fatty Acid Metabolism

**Figure 5:**
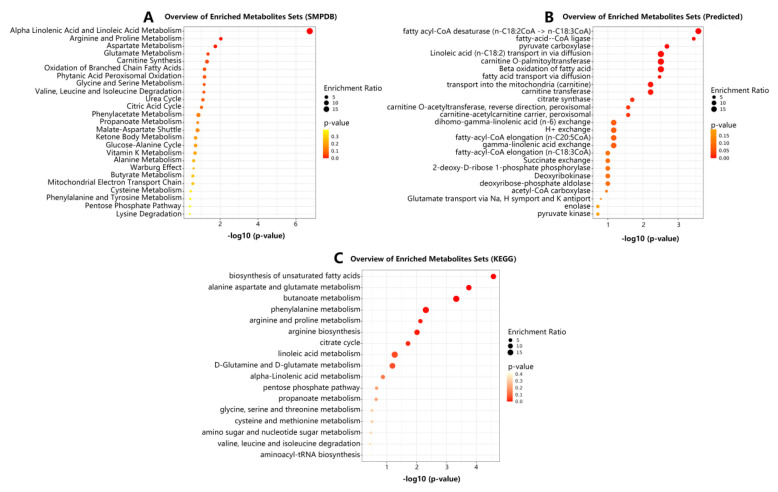
Pathway enrichment analysis of the 39 differential metabolites (DMs). Pathway enrichment analysis of the 39 significant differential metabolites (DMs). The top 25 enriched pathways in pathway-associated metabolite sets (SMPDB, (**A**)), predicted metabolite sets (**B**) and all 17 enriched pathways in KEGG (**C**) are shown.

Pathway enrichment of the 39 DMs was executed via MetaboAnalyst 5.0. Analysis against the SMPDB database significantly enriched 5 pathways (*p* < 0.05), primarily centering on amino acid and fatty acid metabolism (Figure 5A, Supplementary Table S8A). Similarly, predicted metabolite set analysis and KEGG topology mapping consistently highlighted severe perturbations in fatty acid metabolism and various amino acid cascades (Figure 5B,C; Supplementary Table S8B–C). Collectively, the metabolomic data substantiate the transcriptomic findings, confirming that TDF potently disrupts hepatic lipid and amino acid homeostasis. Collectively, the metabolomic data are consistent with the transcriptomic findings and further suggest that TDF exposure is associated with marked disruptions in hepatic lipid and amino acid homeostasis.

### 3.6. Multi-Omics Integration Uncovers Coordinated Suppression of Amino Acid Metabolism and the Citrate Cycle

To systematically define the TDF-driven molecular networks, we performed an integrative analysis intersecting the GSEA (transcriptomics) and metabolomic KEGG pathway enrichments. Strikingly, while no common upregulated pathways were identified (Figure S5A), the intersection revealed four mutually and significantly downregulated pathways: arginine and proline metabolism, alanine, aspartate and glutamate metabolism, phenylalanine metabolism, and the citrate cycle (TCA cycle) (Table 1, Figure S5B).

#### 3.6.1. Arginine, Proline, and Glutamate/Aspartate Networks

**Figure 6:**
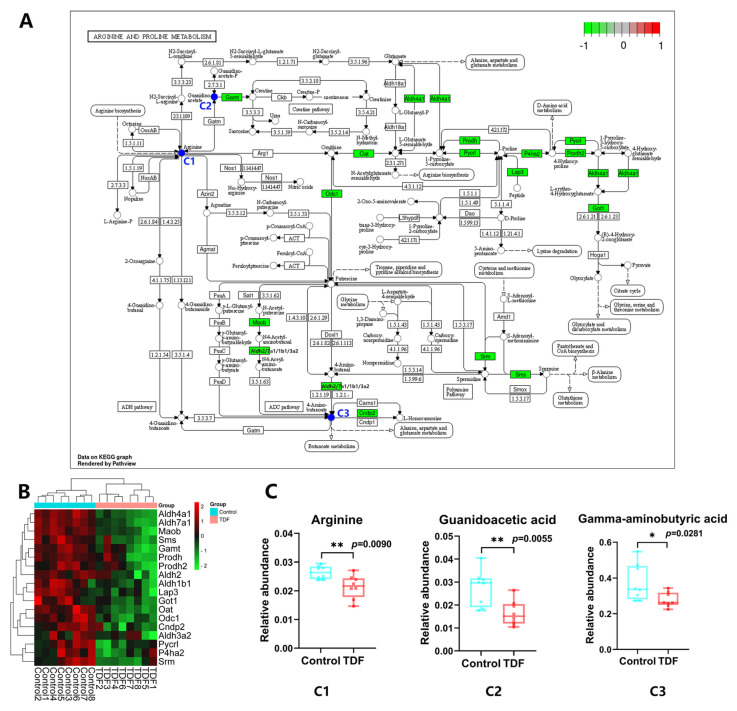
Arginine and proline metabolism pathway. Arginine and proline metabolism pathway. (**A**) The 18 differentially expressed genes (DEGs)and three differential metabolites (DMs) mapped in arginine and proline metabolism pathway. The boxes represent DEGs; green indicates downregulated and red indicates upregulated. The dots represent DMs; blue indicates downregulated and red indicates upregulated. (**B**) Hierarchical clustering heatmap of the 18 DEGs based on Z-score of the transcriptome. (**C**) Relative abundance box plot of the three DMs, arginine (**C1**), guanidoacetic acid (**C2**) and Gamma-Aminobutyric acid (**C3**) based on the metabolome. The data were expressed as mean ± SEM (n = 8); statistical analyses were performed with Student’s *t* tests (**C1**,**C2**) and Mann–Whitney test (**C3**), * *p* < 0.05, ** *p* < 0.01.

**Figure 7:**
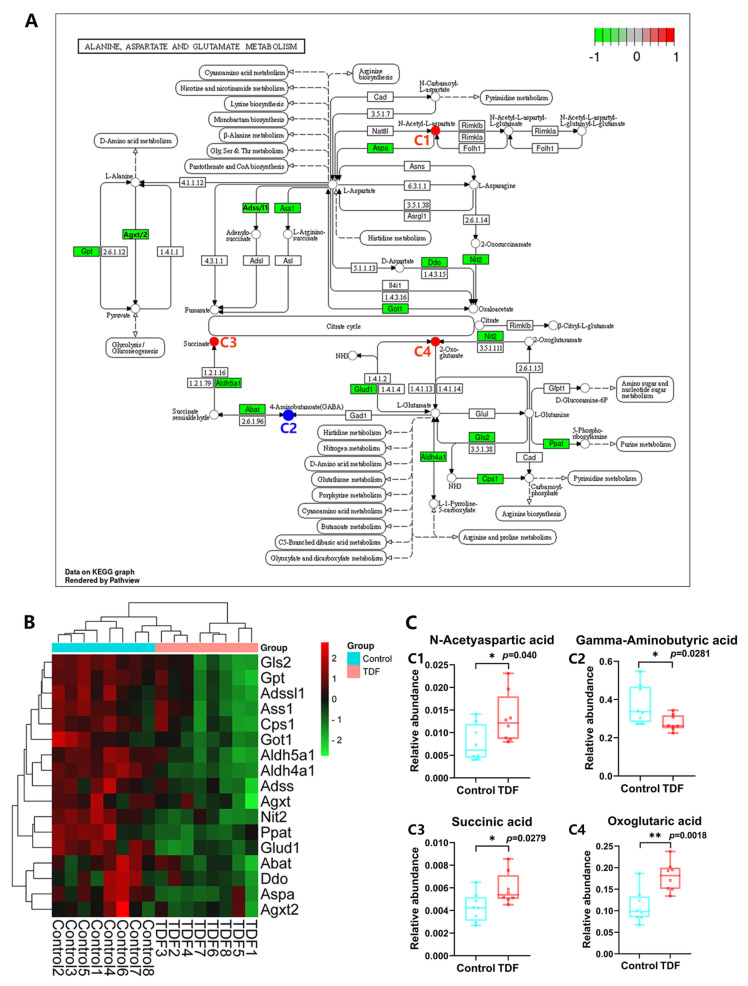
Alanine aspartate and glutamate metabolism pathway. Alanine aspartate and glutamate metabolism pathway. (**A**) The 17 differentially expressed genes (DEGs) and four differential metabolites (DMs) mapped in alanine aspartate and glutamate metabolism pathway. The boxes represent DEGs; green indicates downregulated and red indicates upregulated. The dots represent DMs; blue indicates downregulated and red indicates upregulated. (**B**) Hierarchical clustering heatmap of the 17 DEGs based on Z-score of the transcriptome. (**C**) Relative abundance box plot of the four DMs, N-acetylaspartic acid (**C1**), Gamma-Aminobutyric acid (**C2**), succinic acid (**C3**) and oxoglutaric acid based on the metabolome. The data were expressed as mean ± SEM (n = 8); statistical analyses were performed with Student’s *t* tests (**C1**,**C3**,**C4**) and Mann–Whitney test (**C2**), * *p* < 0.05, ** *p* < 0.01.

The arginine and proline metabolism pathway was significantly suppressed (NES = −1.8914, Figure S5C), driven by the downregulation of 18 key genes (e.g., Aldh2, Cndp2, Got1, Odc1, Oat; Figure 6B). This transcriptional repression was mirrored by significant depletions in downstream metabolites, including arginine, guanidoacetic acid, and Gamma-Aminobutyric Acid (GABA) (Figure 6A,C). Concurrently, the closely interconnected alanine, aspartate, and glutamate metabolism pathway was downregulated (NES = −1.7073, Figure S5D). Here, 17 crucial enzymatic genes (including Ass1, Glud1, Cps1, Gpt, Got1) were uniformly downregulated (Figure 7B). This enzymatic deficit was metabolically reflected by the depletion of GABA, alongside the aberrant accumulation of intermediate metabolites such as N-acetyl-L-aspartic acid, succinate, and oxoglutarate (α-ketoglutarate) (Figure 7A,C).

#### 3.6.2. Phenylalanine Metabolism

**Figure 8:**
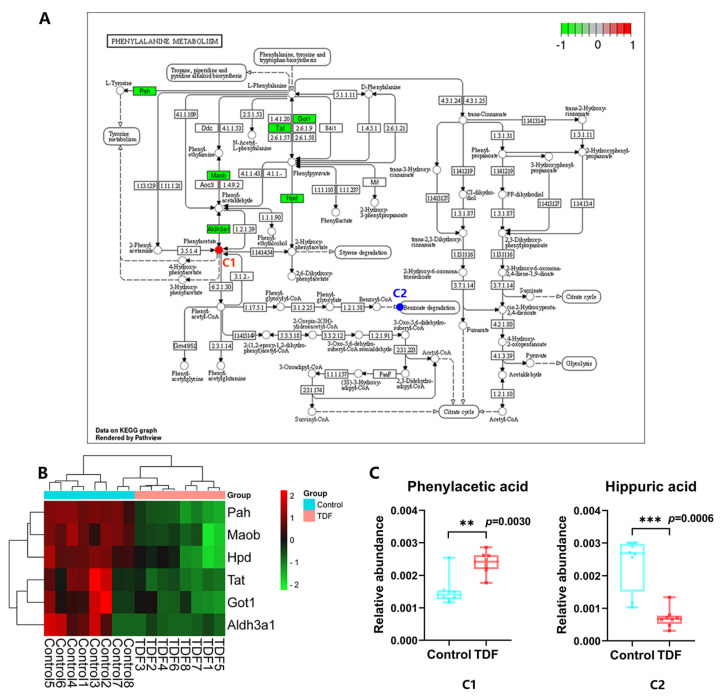
Phenylalanine metabolism pathway. Phenylalanine metabolism pathway. (**A**) The six differentially expressed genes (DEGs) and two differential metabolites (DMs) mapped in phenylalanine metabolism pathway. The boxes represent DEGs; green indicates downregulated and red indicates upregulated. The dots represent DMs; blue indicates downregulated and red indicates upregulated. (**B**) Hierarchical clustering heatmap of the six DEGs based on Z-score of the transcriptome. (**C**) Relative abundance box plot of the two DMs, Phenylacetic acid (**C1**) and Hippuric acid (**C2**), based on the metabolome. The data were expressed as mean ± SEM (n = 8); statistical analyses were performed with Mann–Whitney test, ** *p* < 0.01, *** *p* < 0.001.

TDF administration significantly impaired phenylalanine metabolism (NES = −1.6118, Figure S5E), characterized by the coordinated transcriptional downregulation of 6 central genes, notably Got1, Hpd, Pah, and Tat (Figure 8B). Metabolomically, this disruption manifested as a sharp accumulation of phenylacetic acid (log2FC = 0.7794) and a profound depletion of hippuric acid (log2FC = −1.9869) (Figure 8A,C).

#### 3.6.3. Citrate Cycle (TCA Cycle) Disruption

**Figure 9:**
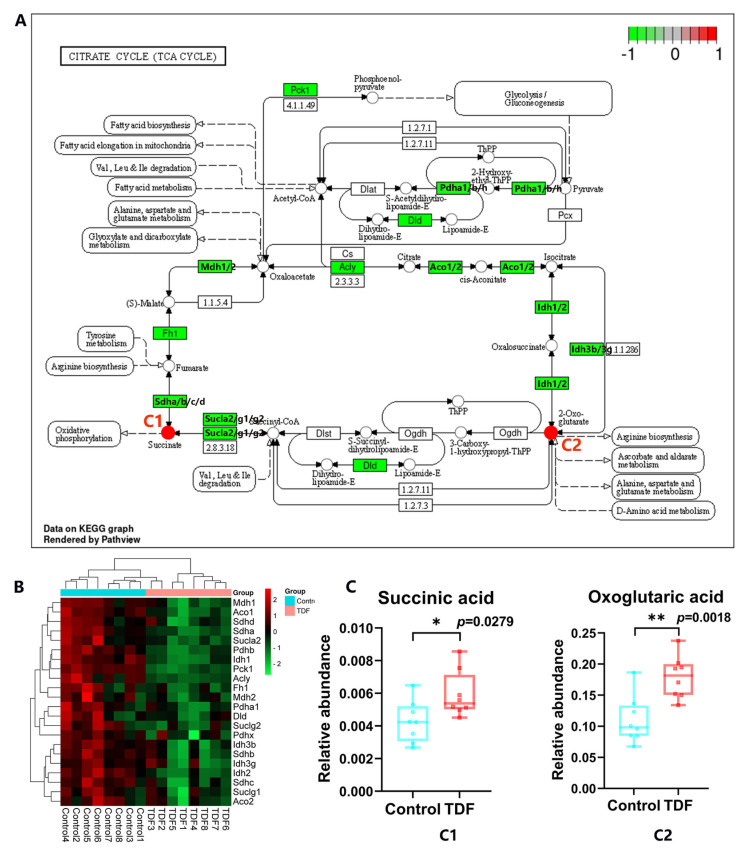
Citrate cycle pathway. Citrate cycle pathway. (**A**) The 22 differentially expressed genes (DEGs) and two differential metabolites (DMs) mapped in citrate cycle pathway. The boxes represent DEGs; green indicates downregulated and red indicates upregulated. The dots represent DMs; blue indicates downregulated and red indicates upregulated. (**B**) Hierarchical clustering heatmap of the 22 DEGs based on Z-score of the transcriptome. (**C**) Relative abundance box plot of the two DMs, Succinic acid (**C1**) and Oxoglutaric acid (**C2**) based on the metabolome. The data were expressed as mean ± SEM (n = 8); statistical analyses were performed with Student’s *t* tests, * *p* < 0.05, ** *p* < 0.01.

Central energy metabolism was markedly inhibited, with the citrate cycle exhibiting significant pathway-level suppression (NES = −1.5452, Figure S5F). We identified 22 core TCA cycle genes that were heavily downregulated by TDF, including crucial node enzymes such as Idh1/2/3 (isocitrate dehydrogenases), Sdh a/b/c/d (succinate dehydrogenases), Mdh1/2 (malate dehydrogenases), and Pdha1/b/x (pyruvate dehydrogenase complex) (Figure 9B). Consequent to the downregulation of these respiratory enzymes, targeted metabolomics revealed a paradoxical accumulation of succinic acid and oxoglutaric acid (Figure 9A,C), strongly suggesting a metabolic bottleneck and impaired flux through the TCA cycle.

Taken together, this robust multi-omics integration provides compelling evidence that TDF administration directly represses core amino acid catabolism and central carbon metabolism in the virus-free liver. Taken together, this multi-omics integration suggests that TDF administration is associated with reduced expression of core amino acid catabolism and central carbon metabolism pathways in the virus-free liver.

## 4. Discussion

While the primary clinical objective of tenofovir disoproxil fumarate (TDF) therapy is the potent suppression of HBV DNA replication, complete eradication of the virus remains a formidable challenge due to the persistence of episomal covalently closed circular DNA (cccDNA) [18]. Consequently, long-term administration of nucleotide analogs is routinely required. Interestingly, accumulating clinical evidence suggests that TDF is not merely a virostatic agent but exerts profound, virus-independent pleiotropic effects on the liver [19]. Compared to entecavir (ETV), TDF has been increasingly associated with unique advantages in immunomodulation, the regression of hepatic fibrosis, and potentially a more pronounced reduction in hepatocellular carcinoma (HCC) risk [5,8,9,10,20,21]. However, the underlying molecular mechanisms driving these extra-antiviral pharmacological properties have remained largely elusive. By utilizing a virus-free wild-type mouse model to isolate direct drug effects from immune responses triggered by viral clearance, our integrated multi-omics study provides a detailed atlas of hepatic transcriptional and metabolic reprogramming associated with long-term TDF administration. By utilizing a virus-free wild-type mouse model to isolate direct drug effects from immune responses triggered by viral clearance, our integrated multi-omics study provides, for the first time, a comprehensive landscape of TDF-induced hepatic transcriptional and metabolic reprogramming.

The most striking finding of the present study is the distinct dichotomy correlated with induced by long-term TDF exposure: a broad transcriptional upregulation of immune and inflammatory responses coupled with a profound suppression of fundamental metabolic networks, particularly amino acid metabolism and the citrate (TCA) cycle. The liver is the central hub for systemic amino acid homeostasis, managing protein synthesis, detoxification (urea cycle), and energetic anaplerosis [22,23]. The comprehensive downregulation of these pathways sheds new light on the cellular mechanisms underlying TDF’s clinical profile.

Specifically, the significant suppression of the arginine and proline metabolism pathway provides a compelling molecular rationale for the clinical observation that TDF potently ameliorates liver fibrosis. Specifically, the significant suppression of the arginine and proline metabolism pathway suggests a potential molecular basis for the clinical observation that TDF exposure is associated with reduced liver fibrosis. Proline is a critical building block for collagen synthesis, the hallmark of extracellular matrix deposition by activated hepatic stellate cells (HSCs) [10,24,25]. The TDF-induced transcriptional repression of key enzymes in this pathway (e.g., Aldh2, Aldh4a1, Oat), accompanied by the depletion of arginine and GABA, suggests that TDF creates a metabolic microenvironment unfavorable for active fibrogenesis. By restricting the availability of fibrogenic amino acids, TDF may directly attenuate HSC activation and collagen deposition, independent of its antiviral efficacy. The transcriptional repression of key enzymes in this pathway (e.g., Aldh2, Aldh4a1, Oat) following TDF exposure, accompanied by the depletion of arginine and GABA, suggests a metabolic microenvironment that may be less favorable for active fibrogenesis. By limiting the availability of fibrogenic amino acids, TDF exposure could potentially attenuate HSC activation and collagen deposition, though this remains to be functionally validated [24].

Furthermore, the coordinated downregulation of alanine, aspartate, and glutamate metabolism, along with phenylalanine catabolism, highlights a broad attenuation of hepatic anaplerotic flux [26,27]. Transaminases such as Got1 and Gpt are essential for transferring amino groups and feeding carbon skeletons into the central energy pathways. The TDF-driven downregulation of these enzymes likely throttles the hepatic energy supply and alters the delicate balance of neurotransmitter precursors (such as the observed depletion of GABA and accumulation of N-acetyl-L-aspartic acid). Additionally, the robust depletion of hippuric acid, a uremic toxin derived from phenylalanine metabolism, implies a potential shift in gut–liver axis metabolism or an alteration in hepatic detoxification capacities under prolonged TDF exposure.

Perhaps the most mechanistically insightful discovery is the profound disruption of the citrate (TCA) cycle. TDF, as a nucleotide analog, has historically been associated with mild mitochondrial impairment [15,28,29,30,31]. Our omics data reveal a systemic downregulation of core respiratory genes (Idh, Sdh, Mdh) and a paradoxical accumulation of succinic acid and oxoglutaric acid (α-ketoglutarate). This signature strongly indicates a “metabolic bottleneck” within the mitochondria. In the emerging field of immunometabolism, the accumulation of TCA cycle intermediates—specifically succinate—acts as a potent danger signal that stabilizes HIF-1α, thereby reprogramming local macrophages toward an activated, pro-inflammatory state [29,32]. This metabolic rewiring elegantly bridges the gap between our two major observations: the suppression of the TCA cycle (resulting in succinate accumulation) may be the precise biochemical trigger for the upregulated immune responses observed in the transcriptome. This mechanism perfectly aligns with recent studies demonstrating TDF’s ability to induce innate immune signaling and IFN-λ3 production. This metabolic pattern suggests a potential link between the two major observations in this study. Suppression of the TCA cycle, accompanied by succinate accumulation, may contribute to the upregulated immune responses observed in the transcriptome, though the directionality of this relationship remains to be tested. This observation is consistent with recent studies reporting TDF-associated induction of innate immune signaling and IFN-λ3 production [8].

Limitations and Future Directions. While this study provides novel molecular insights, several limitations warrant acknowledgment. While this study provides novel insights, several limitations warrant acknowledgment. First, by employing healthy wild-type mice, we successfully isolated the direct pharmacological effects of TDF, but these findings need to be cross-validated in preclinical models of CHB or liver fibrosis to assess their behavior in a diseased microenvironment. Second, although transcriptomic and predictive metabolomic data indicated significant alterations in lipid and retinol metabolism, precise quantification of these lipid species was beyond the scope of our current targeted metabolomics panel. Future lipidomics and targeted retinoid analyses are highly recommended. Third, we acknowledge that this study relies solely on high-throughput omics data without experimental validation. Future studies are therefore needed to validate the key metabolic genes identified herein (e.g., Idh1, Sdhb, Mdh1, and Got1) in independent animal cohorts or TDF-treated hepatocyte cell lines. These validation efforts should also be extended to disease-relevant models, such as HBV transgenic mice or liver fibrosis models, to assess the translational potential of our findings. Finally, translating these specific metabolic intermediates into predictive clinical biomarkers for TDF efficacy or potential off-target toxicities (such as bone mineral density loss or nephrotoxicity) will require large-scale longitudinal human cohorts [28,33].

## 5. Conclusions

For the first time, this integrated transcriptomic and metabolomic atlas reveals that long-term TDF administration directly induces profound metabolic reprogramming in the liver, independent of viral infection. We demonstrate that TDF exerts its pleiotropic extrahepatic effects by simultaneously upregulating immune signaling and suppressing core amino acid metabolism and the citrate cycle. The resultant inhibition of fibrogenic amino acid pathways and the accumulation of immunomodulatory metabolites (such as succinate) offer a novel mechanistic framework explaining TDF’s clinical superiority in fibrosis regression and immune activation. Key nodal genes and accumulated intermediates identified herein represent promising therapeutic targets and potential biomarkers for optimizing individualized anti-HBV strategies. This multi-omics atlas links long-term TDF administration to hepatic immune activation and metabolic suppression in the absence of viral infection, offering a potential framework for understanding TDF’s effects on fibrosis and immunity, and identifying candidate biomarkers and therapeutic targets for CHB.

## Figures and Tables

**Table 1:** The four intersection KEGG pathways between transcriptomics and metabolomics.

KEGG Pathway	Transcriptomics			Metabolomics	
	Core/Size	NES	NOM *p*	Count	*p*-Value
arginine and proline metabolism	18/47	−1.8914	0.00196	3	0.00748
alanine aspartate and glutamate metabolism	17/31	−1.7073	0.01167	4	0.00018
phenylalanine metabolism	6/18	−1.6118	0.01883	2	0.00493
citrate cycle	22/31	−1.5452	0.04457	2	0.0195

Size: number of genes enriched on a certain pathway. Core: number of genes critical for the direction of enrichment of a certain pathway. NES: normalized enrichment score. NOM *p*: normalized *p*-value. Count: number of metabolites enriched on a certain pathway.

## Data Availability

All data needed to understand and assess the conclusions of this research are available in the main text and Supplementary Materials. Raw datasets supporting the findings of this study are available from the corresponding author on reasonable request. The raw data of RNA-seq were translated into FASTQ format and then compressed into .gz files to be deposited at the National Library of Medicine database with accession number PRJNA763152.

## Supplementary Materials

The following supporting information can be downloaded at: https://www.mdpi.com/article/10.3390/life16061017/s1, Figure S1. Flowchart of this research. Figure S2. Principal component analysis of transcriptome. Figure S3. Effect of TDF on basal parameters of wild-type mice. Figure S4. Overview of metabolomics data profiles. Figure S5. Integrated analysis of significant KEGG pathways in transcriptome and metabolome. Table S1. Differentially expressed genes (DEGs) between TDF and control groups. Table S2. GO and KEGG enrichment analysis of DEGs. Table S3. GSEA of DEGs. Table S4. Detailed list of identified metabolites. Table S5. Summary of significantly altered metabolites. Table S6. OPLS-DA and univariate analysis of metabolomics data. Table S7. Key differential metabolites. Table S8. SMPDB and predicted pathway enrichment of metabolomics data. Table S9. Integrated transcriptomic and metabolomic pathway analysis (GSEA and KEGG).
