# Supplementary material for: Multi-Omics Integration Uncovers That Tenofovir Disoproxil Fumarate Is Linked to Hepatic Metabolic Reprogramming Independent of Viral Infection
Source: Life (Basel). 2026 Jun 17;16(6):1017. doi: 10.3390/life16061017 (PMC13301855; doi:10.3390/life16061017)
Supplement: Supplementary file 1 [file life-16-01017-s001.zip › Supplementary material.pdf]

Table of Contents

S1. Transcriptomic analysis

S1.1 Sample selection for transcriptome analysis

S1.2 The filtering and processing of raw data

S1.3 Functional enrichment analyses of transcriptome

S2. Metabolomic analysis

S2.1 Chemicals and reagents

S2.2 Sample preparation

S2.3 UPLC-MS/MS instrument settings

S2.4 Processing and visualization of data

Figure S1. Flowchart of this research

Figure S2. The principal component analysis of transcriptome

Figure S3. The effect of TDF on basal parameters of wild-type mice

Figure S4. Overview of metabolomics data profiles

Figure S5. Integrated analysis of significant KEGG pathways in transcriptome and metabolome

S1.1 Sample selection for transcriptome analysis. Livers from 12 mice in each group were selected for transcriptomic analysis. The RNA was sequenced using the DNBseq platform in BGI (Shenzhen, China), 9 samples in the TDF (T1-T9) group and 11 samples (S1-S11) in the Control group meeting the requirements after quality control, ultimately. The raw data were translated into FASTQ format and then compressed into .gz

files to be deposited at the National Library of Medicine database with accession number PRJNA763152. We eliminated S9, S10, and S11 from the Control group and T1 from the TDF group based on the principal component analysis (Supplementary Fig. S2). Thus, eight samples from each group were finally included in this transcriptomic analysis (renumbered as T1-T8, and S1-S8).

S1.2 The filtering and processing of raw data. Low-quality reads, adaptor reads, and reads with>10% unknown bases (poly-N) were removed from raw data to obtain high-quality "clean" reads for subsequent analyses. The Q20 (percentage of bases with a quality value ≥ 20) and Q30 content of clean data were also calculated. Clean reads were mapped to the Mus musculus reference genome (GRCm38.p6) using HISAT2. Expression was calculated by RSEM and represented in fragments per kilobase per million (FPKM) reads.

S1.3 Functional enrichment analyses of transcriptome. In the results of the DAVID database (https://david.ncifcrf.gov/home.jsp) analysis, terms with false discovery rate (FDR, Q) < 0.05 and gene count ≥ 2 were considered significant. In the results of the Metascape database (https://metascape.org/gp/index.html#/main/step1) analysis, terms with Q < 0.01, minimum count of 3 were considered significant. In the GSEA analysis, the gene lists of KEGG and Biological Process (BP) were download from website (http://ge-lab.org/gskb/). As a metric for ranking

genes in GSEA, the absolute signal to noise value of gene expression was used, and other parameters were set to default values. Terms with normalized p-value < 0.05, absolute value of normalized enrichment score (NES) ≥ 1 were considered significant.

S2.1 Chemicals and reagents. All of the 300 standards were obtained from Sigma-Aldrich (St. Louis, MO, USA), Steraloids Inc. (Newport, RI, USA) and TRC Chemicals (Toronto, ON, Canada). All the standards were accurately weighed and prepared in water，methanol, sodium hydroxide solution, or hydrochloric acid solution to obtain individual stock solution at a concentration of 5.0 mg/ml. Appropriate amount of each stock solution was mixed to create stock calibration solutions.

Formic acid was of analytical grade and obtained from Sigma-Aldrich (St. Louis, MO, USA). Methanol (Optima LC-MS), acetonitrile (Optima LC-MS), and isopropanol (Optima LC-MS) were purchased from Thermo-Fisher Scientific (FairLawn, NJ, USA). Ultrapure water was produced by a Mill-Q Reference system equipped with a LC-MS Pak filter (Millipore, Billerica, MA, USA).

S2.2 Sample preparation. Each liver tissue sample (~10mg) that was harvested and stored in an Eppendorf Safelock microcentrifuge tube, was mixed with 10 pre-chilled zirconium oxide beads and 20μL of deionized water. The sample was homogenated for 3 minutes and 150μL of

Methanol containing internal standard was added to extract the metabolites. The sample was homogenated for another 3 minutes and then centrifuged at 18000g for 20 minutes. Then the supernatant was transferred to a 96-well plate. The following procedures were performed on a Biomek 4000 workstation (Biomek 4000, Beckman Coulter, Inc., Brea, California, USA). 20μL of freshly prepared derivative reagents was added to each well. The plate was sealed and the derivatization was carried out at 30°C for 60 min. After derivatization, the sample was evaporated for 2h. 400μL of ice-cold 50% methanol solution was added to reconstitute the sample. Then the plate was stored at -20°C for 20 minutes and followed by 4000g centrifugation at 4 °C for 30 minutes. 135μL of supernatant was transferred to a new 96-well plate with 15μL internal standards in each well. Serial dilutions of derivatized stock standards were added to the left wells. Finally, the plate was sealed for LC-MS analysis.

S2.3 UPLC-MS/MS instrument settings.

UPLC-MS/MS instrument settings	
**UPLC**	
**Column**	ACQUITY UPLC BEH C18 1.7 µM VanGuard pre-column (2.1×5 mm) and ACQUITY UPLC BEH C18 1.7 µM analytical column (2.1 × 100 mm)
**Column Temp. (°C)**	40
**Sample Manager Temp. (°C)**	10
**Mobile Phases**	A=water with 0.1% formic acid; and B=acetonitrile / IPA (90:10)
**Gradient Conditions**	0-1 min (5% B), 1-12 min (5-80% B), 12-15 min (80-95% B), 15-16 min (95-100%B), 16-18 min (100%B), 18-18.1 min (100-5% B), 18.1-20 min (5% B).
**Flow Rate (mL/min)**	0.40
**Injection Vol. (µl)**	5.0
**MASS SPECTROMETER**	
**Capillary (Kv)**	1.5 (ESI+), 2.0 (ESI-)
**Source Temp (°C)**	150
**Desolvation Temp (°C)**	550
**Desolvation Gas Flow (L/Hr)**	1000

S2.4 Processing and visualization of data. Orthogonal partial least square discriminant analysis (OPLS-DA) of multivariate statistical analyses and univariate statistical analyses (including student t-test, Mann-Whitney-Wilcoxon) were performed by statistical analysis software packages in R studio for further data processing and visualization. The quality of the OPLS-DA models was evaluated based on R^2X or R^2Y and Q^2Y values (R^2Y: provides an estimate of how well the model fits the Y data, Q^2Y: provides an estimate of how well the model predicts the Y data). The cumulative values of R^2Y and Q^2Y approaching 1.0 indicate a reliable model with a satisfactory predictive ability.

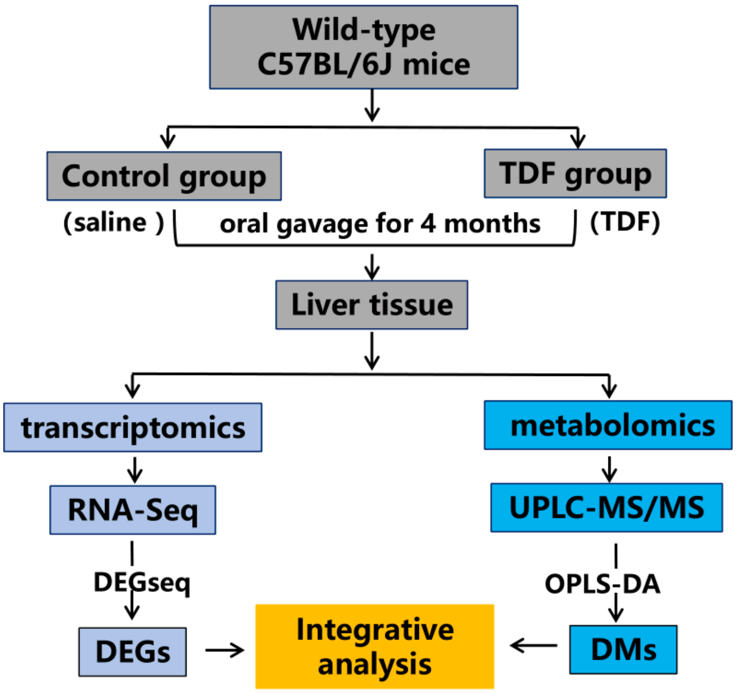

Figure S1. Flowchart of this research. DEGs: differentially expressed genes. UPLC-MS/MS: ultra-performance liquid chromatography coupled to tandem mass spectrometry. OPLS-DA: Orthogonal partial least square discriminant analysis. DMs: differential metabolites.

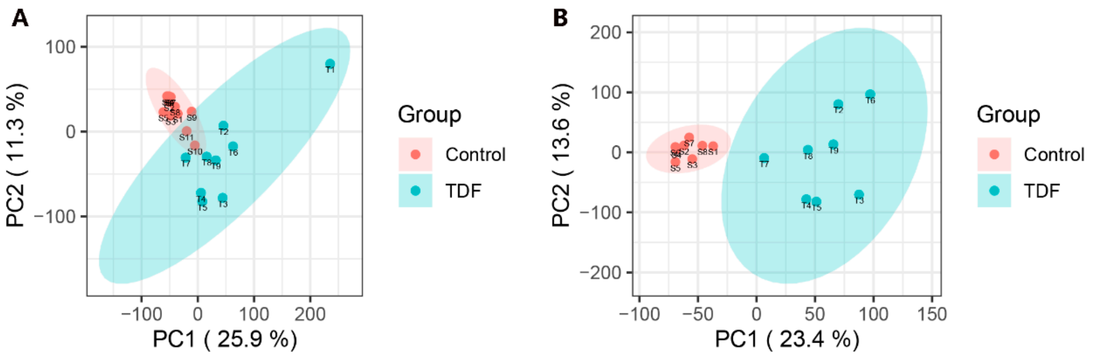

Figure S2. Principal component analysis (PCA) was performed with RNA-Seq data. (A) PCA analysis for all samples, n=11 in the Control group, n=9 in the TDF group. (B) PCA analysis after sample exclusion, n=8 in each group. The S9, S10, S11 in the Control group and T1 in the TDF group were excluded.

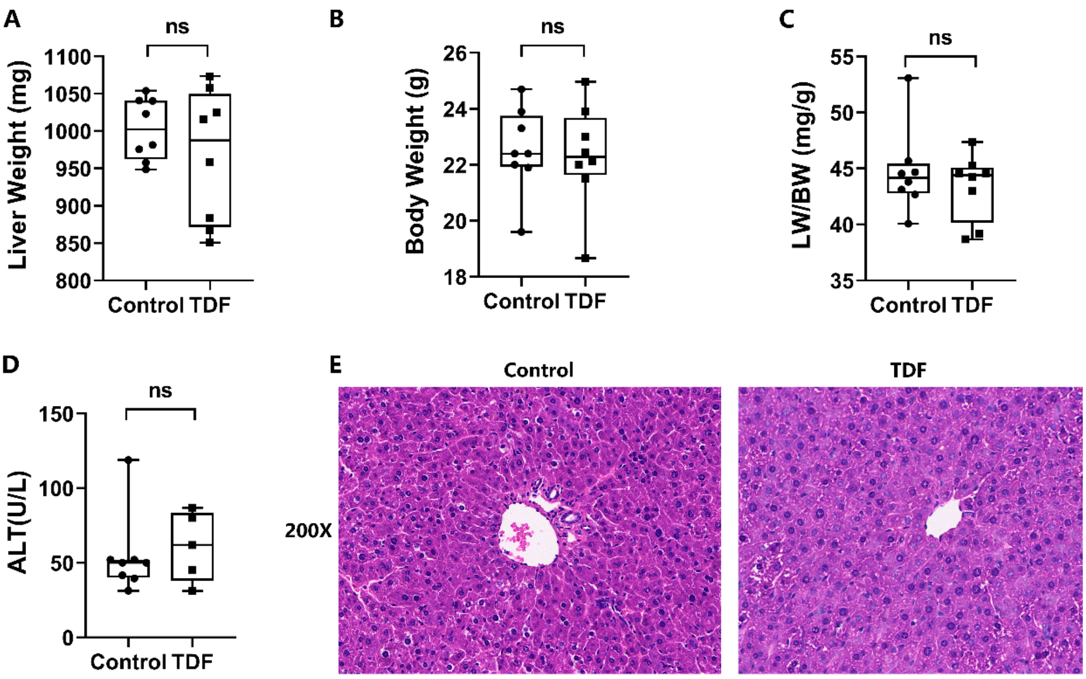

Figure S3. The effect of TDF on basal parameters of wild-type mice. The liver weight (A) and body weight (B) of mice was measured after treatment, n=8. (C) The hepatic index (liver weight/body weight, LW/BW) was calculated, n=8. (D) Plasma ALT level was detected, n=8 in the Control group, n=5 in the TDF group. (E) HE staining of liver sections, 200X. Data were presented as mean ± SEM, statistical analyses were performed with Student' s t tests, NS: No significant.

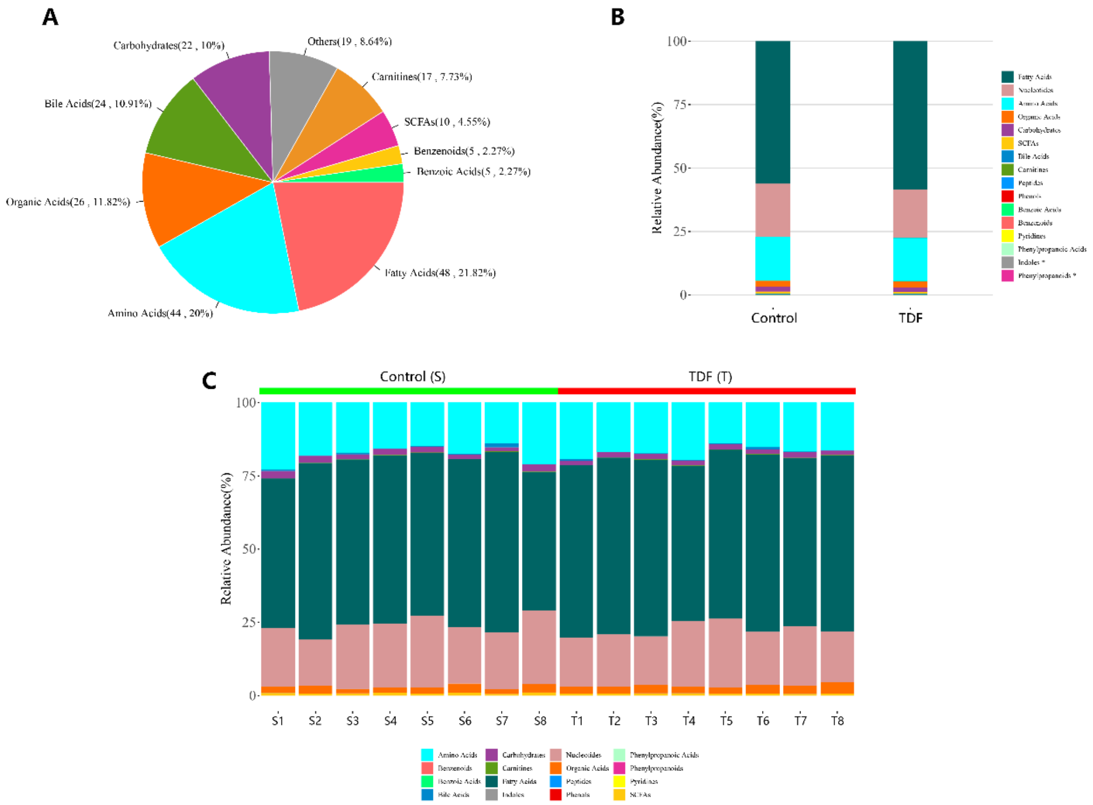

Figure S4. Overview of metabolomics data profiles. (A) The proportion of identified metabolite classes in all samples. (B) The relative abundance of each metabolite classes in different groups. (C) The relative abundance of each metabolite classes in each sample.

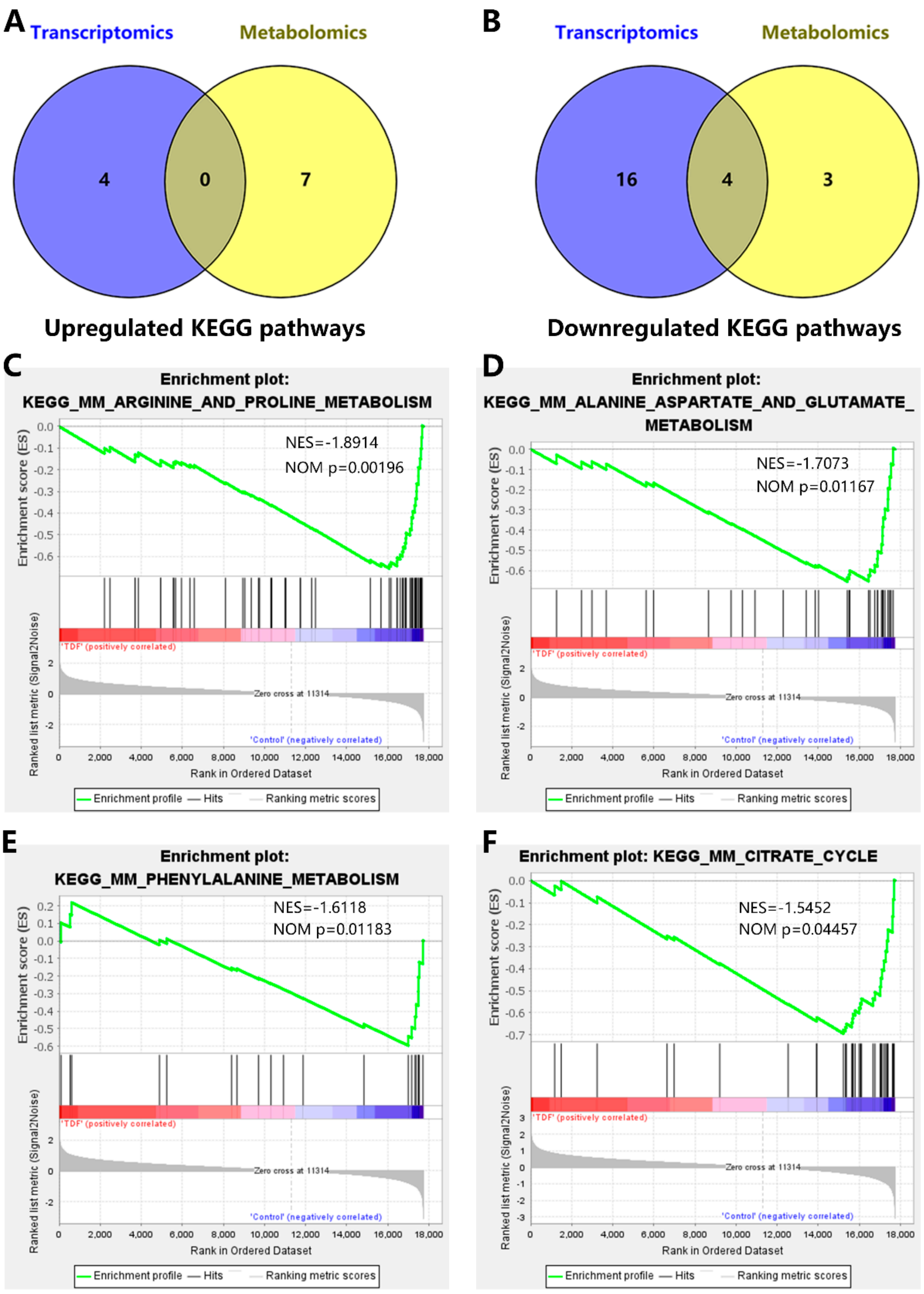

Figure S5. Integrated analysis of union significant upregulated (A) and downregulated (B) KEGG pathways in transcriptome and metabolome. Gene set enrichment analysis (GESA) results of "arginine and proline metabolism" (C), "alanine aspartate and glutamate metabolism" (D), "phenylalanine metabolism" (E), and "citrate cycle" (F).
